# Sociodemographic and Pre-Linguistic Factors in Early Vocabulary Acquisition

**DOI:** 10.3390/children8030206

**Published:** 2021-03-09

**Authors:** Elisabet Serrat-Sellabona, Eva Aguilar-Mediavilla, Mònica Sanz-Torrent, Llorenç Andreu, Anna Amadó, Miquel Serra

**Affiliations:** 1 Psychology Department, Universitat de Girona, 17004 Girona, Spain; anna.amado@udg.edu; 2 Applied Pedagogy and Educational Psychology, Institute of Research and Innovation in Education (IRIE), Universitat de les Illes Balears, 07122 Palma, Spain; 3 Psychology Faculty, Universitat de Barcelona, 08035 Barcelona, Spain; monicasanz@ub.edu (M.S.-T.); miquel.serra@ub.edu (M.S.); 4 Psychology and Education Science Studies, Universitat Oberta de Catalunya, 08018 Barcelona, Spain; landreub@uoc.edu

**Keywords:** MacArthur-Bates CDI, Catalan, lexical spurt, sex, birth order, birth weight, parental education, imitation, gestures, comprehension

## Abstract

Here, we studied the beginnings of language development, jointly assessing two groups of precursors, sociodemographic and pre-linguistic, that have previously been studied separately. Thus, the general objective of this study was to explore which factors best explained the acquisition of initial expressive vocabulary. The sample consisted of 504 participants from Catalan-speaking homes with ages ranging between 10 and 18 months. The data were obtained through the MacArthur–Bates Communicative Development Inventories (MCB-CDIs). Vocabulary development shows a lexical spurt at 17 months. Regression analyses show that pre-linguistic factors have more explanatory power of than sociodemographic ones. Within the sociodemographic variables, age, birth order and birth weight explain part of the vocabulary variance. With respect to pre-linguistic variables, imitation, late gestures and phrase comprehension are predictors of the initial vocabulary acquisition. Specifically, imitation and late gestures were the pre-linguistic behaviours that made it possible to distinguish between children with higher and lower levels of vocabulary. We discussed these findings in relation to their relevance for language acquisition and for the early assessment of linguistic competence.

## 1. Introduction

The aim of this work was to study the influence of factors traditionally considered related to initial language acquisition. Until now, personal, and sociodemographic factors have often been considered separately from pre-linguistic factors, but here we present a joint evaluation of how these two factors affect initial language acquisition.

The cognitive abilities of children and their relationship with the environment are two variables that are in constant interaction and are responsible for determining a child’s communicative, cognitive, and affective development. With regard to communicative development, typically developing children have already discovered patterns of meaning in speech by the end of their first year of life [1,2]. It is around this time that children start to discover connections between language and the world around them [3,4]. Any disruption at this initial stage will affect a child’s linguistic development as well as other related forms of development and subsequent learning experiences [5].

Thus, it is during the first and second years of life that the foundations for communicative and linguistic development are laid. While this kind of development is important enough in itself, it also has repercussions for other aspects of development. Accordingly, our study recognizes the relevance of exploring and evaluating this initial stage of language development and the factors that influence it. To examine some of these factors more closely, we first review the sociodemographic variables that have been considered to play a role in language development, and then examine some of the pre-linguistic skills also considered relevant during initial language acquisition.

### 1.1. Sociodemographic Variables Related to Language Acquisition

Previous studies have shown that some demographic, personal, and social variables are related to language development. These include sex, birth weight, history of ear infections, birth order, parents’ level of education, and parents’ education level, and their socio-cultural and economic status.

Although the male sex has traditionally been linked with lower language abilities [6] and a greater prevalence of language difficulties [7], there is still no evidence of any biological causes that explain this [8]. Besides, more recent and large-scale studies have found a more balanced ratio and fewer differences between boys and girls [9]. With respect to brain differences related to language development, a systematic review by Etchell et al. [8] showed that brain differences between the sexes may be more prominent during certain developmental stages but are negligible in other stages, suggesting that such differences are not as significant as previously thought. However, most studies that evaluate linguistic performance between boys and girls during language acquisition have found differences between the sexes. For example, Huttenlocher et al. [10] found sex differences related to lexical growth in children from 22 to 26 months. Galsworthy et al. [11] found that 2-year-old girls outperformed boys in verbal development. Various large-scale studies conducted using the MacArthur–Bates Communicative Development Inventories (CDIs) [12] found early sex differences that increase with age in children from 8 to 36 months, both from English and non-English-speaking backgrounds, with girls outperforming boys in early communicative gestures, vocabulary, and word combinations [13,14,15,16]. However, other studies also conducted using the CDIs, such as Kovačević et al. [17], Jackson-Maldonado et al. [18], and Berglund and Eriksson [19], failed to find differences in language development between boys and girls. 

Birth weight has also been related with language development. Children born with a low weight are at higher risk of experiencing language problems [20,21,22]. The risk of language problems increases the lower birth weight was [23]. The relation between birth weight and language development is also influenced by other factors such as medical complications and born prematurely [22]. Thus, healthy children with weights upper than 1900 gr. at born show language abilities adequate to their gestational age [24]. Contrary, a weight lower than 1500 gr. at born is related with a higher incidence of medical complications such as conductive hearing loss, which can interfere with language acquisition and is considered a risk factor for deafness [25]. 

Despite the fact that hearing loss is a clear factor in delaying spoken language development, the association between otitis (i.e., inflammation that occurs within the middle-ear cavity and causes mild-to-moderate hearing loss) and language development is not so clear [26]. Although traditionally [27] otitis has been linked with a major risk of suffering language development delays, a review by Roberts et al. [28] found that otitis media with effusion may not be a substantial risk factor for later speech and language development in typically developing children. Despite these results, otitis is still considered a risk factor for language delay in clinical settings [29]. 

Birth order is another variable that has traditionally been linked with language development, with firstborns exhibiting better language abilities than later-born children [13]. In this regard, Fenson et al. [14] noted significant negative correlations between birth order and gestures, vocabulary production, Mean Length of Utterances (MLU) and word combinations measured using the CDI. Berglund et al. [13] found better vocabulary comprehension and production in firstborns assessed at 18 months using the Swedish Early Communicative Development Inventories. Firstborn advantage in language acquisition has not only been found using parent inventories, but also through direct observations. For example, by observing natural language, Hoff-Ginsberg [30] showed that firstborns had an advantage in lexical and grammar development at 18 to 29 months. Nevertheless, the evidence of quantitative differences between firstborn and later-born infants is inconclusive; for example, Pine [31] found that firstborns reached the 50-word milestone earlier than secondborns, but found no differences in reaching the 100-word milestone. Oshima-Takane et al. [32] reported no differences in the MLU, number of intelligible utterances, total vocabulary (types) and total number of words (tokens) between firstborns and secondborns at 21 and 24 months, but an advanced use of pronoun productions was found in secondborns.

Some social aspects have also been linked with language development. Of particular interest to researchers is the relationship between socioeconomic status (SES) and language outcomes, which has been found to be incredibly convoluted and complex [30,33,34,35,36,37]. For example, socioeconomic status is decisive for other variables that can affect language development such as the family home, neighbourhood, child’s school, and the resources to which he/she has access [38]. Besides, the relation between SES and language is mediated by other variables, such as parent’s educational level, cultural differences and the linguistic input that the child receives [38,39,40]. Previous studies have highlighted the importance of quantity of input exposure, but most acknowledge that the quality of language that the child is exposed to is more salient [41,42]. In this sense, some studies [33,38,43] have found that a higher maternal education could be a protective factor against language difficulties and a predictor of better child language development. For example, Hirsh-Pasek et al. [36] show in low incoming families that maternal education is related with sensitive parenting, the quantity of language input the child received, the quality of communicative interactions (e.g., use of routines and rituals) and child expressive language. Accordingly, previous studies have found maternal educational level related to early language development, as measured by the MacArthur Communicative Development Inventories [44,45], although others have failed to find this relationship when linguistically, culturally, and developmentally appropriate instruments are used [46].

### 1.2. Pre-Linguistic Factors

During the pre-linguistic period, children’s language develops as follows: (a) a focus on the sounds of speech; (b) understanding first words; (c) communicating needs through language; (d) random vocalizations; and (e) uttering familiar speech sounds. At this time, they also progress from an initial multimodal perception of their postnatal environment to attaching symbolic representations and references to actions, objects, and significant people. While studying this period, researchers have focused mainly on intentional communication, vocalizations, and gestures as precursors and possibly facilitators of a child’s first words [47,48].

#### 1.2.1. First Signs of Understanding

Over recent decades, many studies have focused on how babies know and comprehend aspects of the language in early development and before they say their first words. These studies show how babies recognize the voice of their mothers [49,50] and are able to discriminate their language from others [51]. Saffran et al. [52,53] showed how children are able to extract regularities of speech and recognize parts and patterns in the flow of speech heard. These authors found that after just 2 minutes of exposure, 8-month-old infants could extract words embedded in a continuous stream of spoken artificial language. This type of learning has been called statistical language learning [52]. It has also been shown that crying [54] and the intonation of babbling vary depending on the language of exposure [55], which is a sign that children are attentive and analyzing the speech to which they are exposed and trying to approximate those patterns.

Before they say their first words, babies can also understand the pragmatic intention of adults from highly context-dependent situational clues. At 9 months, children understand some words and expressions of adults. They react to their name or respond in some way to very specific words or expressions spoken with a certain intonation and in repetitive or familiar contexts [48,56]. Around the first year of life, children already react to some words or expressions and understand some very simple instructions or phrases related to routines or very familiar situations such as “A dormir” (to bed), “Ja està” (it is over), “Què vols?” (what do you want?) [48,56]. For example, at 12 months, babies would move their head in response to their own name and can begin to understand simple commands or phrases related to routines. At 18 months, they can understand simple commands (one step) such as “Put it here”, “Give me a kiss” or “Say goodbye” [57,58,59]. Additionally, at around the age of 2 years, children use syntactic clues, such as word order, to understand transitive sentences [60,61].

It is possible that the difficulties in understanding spoken language at early ages is one of the main predictors of experiencing a language development disorder later on [62]. In line with this, several studies have found a strong correlation between sentence comprehension in the first 12–18 months and subsequent language level [45,63]. For example, Watt et al. [64] analysed which pre-linguistic skills and behaviours at 1 and 2 years of age predict language abilities at 3 years of age, using communication and symbolic behaviour scales to measure this [65]. The results showed that early comprehension abilities predict subsequent receptive and expressive language outcomes. Several studies have been conducted to assess these early signs of understanding in children with communication or language difficulties. This is the case in a study of children at risk of autistic spectrum disorder (ASD), which observed low scores in the children’s social interest or in responding to theirs names and in understanding initial sentences [66]. Another study conducted with English-speaking infants with and without ASD showed that parents reported fewer sentences understood and fewer gestures produced by 12 months of age in children at risk of ASD measured using the CDI. Luyster et al. [67] reported similar results with “first signs of understanding” and “understanding of phrases” in children with ASD. Finally, Charman et al. [68] also observed, using parental reports, delays in early signs of understanding (e.g., “reacting to mother’s/father’s name”) in children with ASD. In this same line of research, the new conception of Developmental Language Disorders (DLD) includes the presence of comprehension problems between the ages of 2 and 3 years as a factor of early detection, which correlates with the subsequent diagnosis [62].

#### 1.2.2. Imitation

Imitation is another precursor to language development. Verbal imitation, or the repetition of new words or parts of sentences, is a pervasive and innate behavior in early development and is used for diverse functions during language acquisition [69]. One of these functions is to internalize language [70]; in this sense, during our everyday interactions we can see that as children learn language, they spontaneously imitate the speech of those around them. Despite the importance of imitation, few studies have been conducted on its role in language acquisition since the seminal studies by Snow in the 1980s. These first studies focused on verbal imitation and mimicry in the early stages. In accordance with Snow [71], the results of these studies can be placed on a continuum that ranges from the non-contribution of verbal imitation in language development [72,73] to the idea that imitation is at least partially credited for parts of a child’s language development such as the acquisition of vocabulary [74,75,76], grammar [77], morphology and syntax [78,79]. 

With regard to the development of expressive vocabulary, in a recent study conducted by Masur and Olson [80], children who demonstrated more verbal imitations of the language produced by their mothers were found to have a more advanced vocabulary at 17 and/or 21 months. Research carried out with children with atypical development, such as that of Feeley and Jones [81] on children with Down syndrome, or that of Yoder and Layton [82] and Smith et al. [83] on children with ASD, also describe this relationship between verbal imitation and expressive vocabulary.

Studies that deny the contribution of imitation argue that there is considerable individual variance in imitation among children and that only a subgroup of children learn language, or part of it, through imitation. On the other hand, studies that accept a partial contribution of imitation to language development base their assumption on the idea that children imitate syntactic structures when they cannot produce them spontaneously.

Contemporary research has focused primarily on the role of socio-cognitive abilities in verbal imitation, such as understanding the intentions of others or the context in which the sentence is produced. The results of studies conducted by Over and Gattis [84] and Bannard et al. [85] showed that children use the intention perceived in others [84,85] and the functional context of an utterance [85] to imitate a verbal model. 

Finally, a last group of studies provide evidence in favour of verbal over non-verbal imitation in humans and the diversity of purposes of the former. For example, instrumental imitation may have the purpose of (a) transmitting language from one individual to another; (b) engaging in a conversation; and (c) establishing affiliation with others [86,87,88,89]. Beyond these purposes we can add an additional purpose from the study conducted by Matthews et al. [90] that is unique to the verbal domain: (d) facilitating communication, because the conversation becomes more efficient when speakers construct referential pacts.

#### 1.2.3. Gestures and Actions

As several authors have recognized, gestures are one of the most important precursors of language acquisition [91,92]. In this sense, gestures have been considered as behaviours that precede and prepare the emergence of expressive language [93]. 

Some authors have focused on deictic gestures, such as the gesture of pointing, which is considered a precursor of child vocabulary. Nevertheless, more research is needed to establish whether it can be considered a predictor of language development (see [94]), because authors such as McGillion et al. [95] found that the presence of pointing gesture does not predict expressive vocabulary, although the presence of pointing gestures is related to receptive vocabulary. 

The communicative gestures that children make during their first years are not limited to the gesture of pointing. Nelson [96] suggested that gestures and actions, including those integrated in symbolic play, contribute to the development of the representational abilities that are fundamental to language acquisition. A longitudinal study by Cadime et al. [97], with the MacArthur Bates CDI-I, found that gestures predicted vocabulary comprehension at 9, 12, and 15 months, although gestures only predicted expressive vocabulary at 12 months.

Regarding more complex gestures and actions, such as actions that children perform with dolls or through games in which they imitate the actions of adults, it is important to note that these actions can be interpreted as part of symbolic play. In this sense, the relationship between language development and symbolic play has been shown to be robust throughout development [98,99]. Furthermore, despite the fact that there are different interpretations of this relationship, symbolic play can be considered a precursor of language [98].

### 1.3. Present Study

Given the results of the studies reviewed above, several sociodemographic and pre-linguistic factors can influence language acquisition. This study seeks to add to the field by investigating two main questions: 

RQ1: Which of the sociodemographic or pre-linguistic variable(s) studied explain early vocabulary acquisition?

RQ2: Which of the sociodemographic or pre-linguistic variable(s) studied discriminate children with a high level of vocabulary from those with a low level of vocabulary?

The influence of sociodemographic and pre-linguistic factors in early language development would be evident in the comprehension and production of first words. In this study, we focus on vocabulary production, as it is a more valid measure of the MacArthur Bates CD1-I questionnaire in early learners [100]. As we have previously stated, this work aims to study the beginnings of language development, jointly assessing two groups of precursors that have previously been studied separately, that is, sociodemographic and pre-linguistic factors. To ensure that the data are treated collectively rather than separately, data were collected using a single instrument, the MacArthur–Bates CDI, which has been shown to be reliable and valid for child language assessment [14].

Thus, the general objective of this study was to explore which factors best explain the acquisition of initial expressive vocabulary and to what extent they do so. We aimed to describe the course taken in the initial acquisition of expressive vocabulary through the use of parental reports. In this study, this description will allow us to verify that our data conformed to the expected course of vocabulary across the ages studied. Then, we aimed to explore and quantify which factors have the most explanatory power in terms of the acquisition of initial vocabulary, contrasting personal and sociodemographic factors with pre-linguistic factors. Finally, we aimed to measure the contribution of these factors to discriminate children with a high level of vocabulary (>25th percentile) from those who are at risk of suffering delays in their language acquisition. A better understanding of the influence of these aspects on language acquisition is important to design effective assessment tools and interventions. 

## 2. Materials and Methods

### 2.1. Participants

The total sample consisted of 504 participants (259 girls) from Catalan-speaking homes with ages ranging from 10 and 18 months (M age = 14.23; SD = 2.5). Premature children with a weight below 1900 g were excluded due to the medical complications associated with this condition. Table 1 shows the characteristics of the sample, including sociodemographic factors. 

### 2.2. Materials

The data in this study were obtained using the MacArthur–Bates Communicative Development Inventories (MCB-CDIs) adapted for Catalan [101,102]. Specifically, CDI-I was used for this study as it is appropriate for children between the ages of 8 and 18 months. The inventory has two main sections with different sub-sections. 

For the first part, the parents were asked about their child’s first words based on his/her first signs of understanding (the child’s name, “no”, and the names of the parents), how the child understands frequently spoken phrases, the child’s capacity to imitate language, and the list of vocabulary understood and produced. In the second part of the instrument, the parents were asked about the child’s gestures and actions through the use of first gestures of intentional communications, games with adults, turn-taking routines, actions with objects, and symbolic play (e.g., with dolls, imitating adults, and using objects for a different purpose). In the current study, most of the sections of the MCB-CDI-I were considered as independent variables, except the section “Checklist of total vocabulary” that was considered as dependent variable.

The first part of the MCB-CDI-I was divided in several sections as described below. The section “First signs of understanding” contained three items and recorded whether the children reacted when hearing certain words. Specifically, the items in this section referred to whether the child stopped what he/she is doing upon hearing “no” said to him/her; whether he/she responds when called by their name; and whether the child looks around when hearing his/her mother or father be called by name. In the section “Phrases”, which contained 27 phrases or utterances, the number of phrases that the children understood from the section was counted. This section refers to the ability of the children to understand frequently spoken linguistic utterances in speech directed at the children [57,58,59]. Examples of this type of utterance are: “Què és això?” (What is that?), “A dormir” (Go to sleep), “Fes-me un petó” (Give me a kiss), “Quiet!” (Stop!), “Digues adéu” (Say bye-bye), etc. The section “Starting to talk” contained two items (Imitation and Naming), and in this work we only used “Imitation” because “Naming” was considered to be the same as or similar to the “Vocabulary Checklist”. In the “Imitation” section the parents were asked whether the children imitate any words or parts of phrases. The possible answers were “Not yet”, “Sometimes”, and “Often”. The section “Checklist of total vocabulary” contained items from different lexical categories that the child “can understand” or “understand and produce”, such as sound effects and animal sounds, animals (real or toys), vehicles (real or toys), toys, food and drink, clothes, parts of the body, furniture, domestic objects, objects from outside the home and places to visit, people, games, routines and social formulas, actions, times, qualities and attributes, pronouns and possessive and demonstrative pronouns, questions, prepositions, quantifiers, and articles.

The second part of the MCB-CDI-I evaluates gestures and actions and was divided into two main sections, as described below. The first section is called “First gestures and actions”, and contained a sub-section of communicative gestures (e.g., saying “bye” with your hand) and a sub-section of nursery rhymes, children’s songs and routines (e.g., peek-a-boo). The second section is called “Late gestures and actions”, and contained three sub-sections: (1) Performing actions with objects such as the child putting the telephone to his/her ear; (2) playing at being an adult, where the participants are asked about symbolic play activities with a doll (e.g., combing a doll’s hair); and (3) pretending or trying to do adult activities (e.g., pretending to take photos, pretending to sweep, etc.). This section includes many actions or activities of symbolic play. 

Several bibliographic sources on language acquisition show that parental reports are reliable and valid, and represent the linguistic abilities of the children in the short and long term [12,103,104]. It is worth noting that Marchmann and Martínez-Sussman [105] have referred to a high concurrent validity between the development of productive vocabulary measured using the CDI questionnaire and that measured in laboratories. In addition, the MacArthur–Bates questionnaire offers the possibility to analyse the communicative and linguistic development of broad samples of participants, as in our case (see [12]). Specifically, the Catalan version of the MCB-CDI-I has an internal consistency of α = 0.893; a test-retest reliability of α = 0.800 for word comprehension; and a concurrent validity (MCB-CDI-II inventory with display of spontaneous speech) of *r* = 0.577 [102].

### 2.3. Procedure

The participants were recruited through professional and personal contacts of the authors of the adapted questionnaires, as well as through the participation of several child education centres. The CDI-I forms were delivered to the families either personally, in which case they were given instructions on paper that were briefly discussed, or through the early childhood education centres (0–3 years) by giving families an information letter and consent form, with the instructions provided later along with the booklet. The instrument itself contained instructions in each of the sections and the families were explicitly informed, either verbally or through the letter and informed consent, that they should only record the words/usage that their child produced in any variant of the Catalan language, even if there was a mispronunciation. 

The study used the total vocabulary produced by the children as a dependent variable. To calculate the size of each participant’s vocabulary, the total number of words marked by the parents in the “Checklist of total vocabulary section” (one point for each word) was added up. The maximum possible point score was 423 (total number of items in the list).

The information items in the “General information” section of the MCB-CDI-I were used as independent personal and sociodemographic variables—sex, birth order, birth weight, how many ear infections per year, and mother’s and father’s level of education. As for the pre-linguistic independent variables, all sections were scored as indicated in the scoring manual for the instrument. 

A stepwise multivariate regression analysis was performed. Preliminary analyses were conducted to ensure that the assumptions of the multiple regression were met. A logistic regression analysis was also carried out. In this case, the dependent variable was dichotomized based on a child’s normative scores [102]. Participants were grouped according to whether they showed a high level of vocabulary (≥25th) or a low level of vocabulary (<25th). The data were analysed using SPSS v. 23.

## 3. Results

First, curvilinear estimation (a type of regression analysis) was used to determine which model best fit the course of acquisition of expressive vocabulary. Linear, quadratic, and exponential models were chosen for the analysis, as they are appropriate for the field of child development [106].

**Figure 1:**
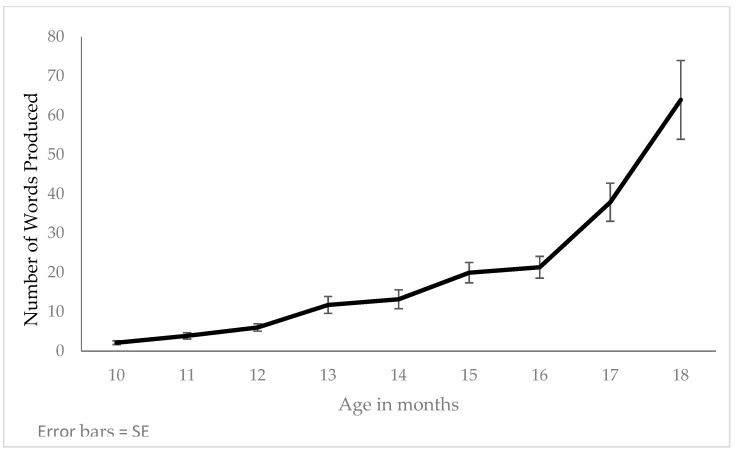
Production of words according to child’s age in months.

It was observed that the vocabulary acquisition process began slowly, increasing gradually until 16 months of age. However, from the age of 17 months, a substantial change in trend was noted (see Figure 1). The children’s vocabulary at this age increases from two words at 10 months to around 20 words on average at 16 months. From this age onwards, and in just two months, the vocabulary increases to over 60 words at 18 months.

Accordingly, the results of the curvilinear estimation reflect the fact that the linear, quadratic, and exponential models adequately conform to the curve. The ANOVA of each model gave the following statistical values: linear (*F*(1.502) = 132.276; *p* < 0.001; *R*^2^ = 0.209), quadratic (*F*(2.501) = 81.451; *p* < 0.001; *R*^2^ = 0.245) and exponential (*F*(1.502) = 281.978; *p* < 0.001; *R*^2^ = 0.360).The model that best fits the data is the exponential model, which has the highest *R*^2^ value and explains more of the variability in the data than the other two models (36% of variance).

### 3.1. Which of the Sociodemographic or Pre-Linguistic Variable(s) Studied Explain Early Vocabulary Acquisition?

A summary of the data for the pre-linguistic variables is shown in Table 2. The significant findings from these data are that most children (88.6%) displayed the three behaviours corresponding to first signs of understanding, a large minority of the sample (40.9%) did not imitate words or parts of phrases, and 59.1% imitate them sometimes or often.

For the second objective of the study, we used a stepwise regression analysis to identify which were the best predictors of vocabulary size. In this regression method, all variables, sociodemographic and pre-linguistic, were considered as predictors, and automatically in each step, the variable accounting for the most proportion of variance was introduced in the model, thus reducing the number of variables in the final model. The significance of the variables in the models, coefficient of determination (*R*^2^) and standardized coefficients (*β*) were used to interpret the significant predictors, proportion of explained variance and the relative weights of each predictor variable, respectively.

Table 3 shows the data from the sixth model generated by the multivariate regression analysis, based on the data from the stepwise method. The regression coefficient is provided with confidence intervals, the standardized score (β) and the statistical significance. Specifically, in the multiple regression analysis, the six predictors explain 65.1% of the variance in the score for expressive vocabulary (*F* = 133.695; *p* < 0.001; adjusted *R*^2^ = 0.651). All the steps that the regression analysis generated can be found in the Appendix A.

Only six of the predictors explained the acquisition of expressive vocabulary in the ages studied, namely, imitation, the understanding of phrases, late gestures and actions, age, birth order, and birth weight. Looking at the standardized scores (β), it can be observed that imitation had the greatest predictive power, followed by late gestures and actions and, to a lesser extent, the understanding of phrases, age, birth order, and birth weight.

### 3.2. Which of the Sociodemographic or Pre-Linguistic Variable(s) Studied Discriminate Children with a High Level of Vocabulary from Those with a Low Level of Vocabulary?

The children were classified according to the dependent variable (expressive vocabulary) based on their normative scores [102]. The cut-off point was applied at the 25th percentile score. Children were grouped according to whether they showed a high level of vocabulary (≥25th percentile) or a low level of vocabulary (<25th percentile). Based on this grouping, a binary logistic regression analysis was then carried out (stepwise method).

A total of 352 children (69.8%) obtained vocabulary scores above the 25th percentile, while 152 children were below this percentile. From this sample, the logistic regression analysis included a total of 428 participants (76 missing values). Table 4 shows that out of the ten possible predictors, only two (imitation and late gestures) are associated with vocabulary scores above the 25th percentile at a significance level of 5%. 

According to Nagelkerke’s determination coefficient, the model explains 37.4% of the variance in the dependent variable. 

To assess whether the predictor variables enabled the discrimination between a high level of vocabulary and a low level of vocabulary, Table 5 shows the specificity and sensitivity of the model generated by logistic regression analysis. The classification table shows that the model has good specificity (83.3%) but low sensitivity (53.9 %). We can therefore interpret from the results that when the predictor variables are present to a greater degree, there is a high probability that children show a high level of vocabulary, with little concern of vocabulary difficulties occurring. However, an absence or limited presence of imitation and late gestures does not discriminate adequately between children with a low or high level of vocabulary, that is, we cannot distinguish whether or not a vocabulary delay would be present when a child shows low levels of imitation and gesture use. 

## 4. Discussion

The results of our study show that both sociodemographic and pre-linguistic variables affect the acquisition of the initial vocabulary, although the latter has more explanatory weight than the former, except for age. In terms of the personal and sociodemographic variables, only the age, birth order and birth weight, in descending order of importance, significantly predicted vocabulary development. With respect to pre-linguistic factors, imitation, late gestures and first sentences comprehension were significant predictors of initial vocabulary production. Thus, demographic factors in conjunction with pre-linguistic ones are useful in explaining the initial vocabulary acquisition with a high amount of variance explained (65.1%). In a detailed analysis, the results of multiple regression and logistic regression indicated that imitation had the greatest explanatory weight. Moreover, the presence of this behaviour can adequately discriminate children with high and low levels of vocabulary. 

### 4.1. Initial Vocabulary

Age is a predictor of vocabulary level, but is not as good as it might seem, as its impact was third in the order of weighting predictors used in this study and was preceded by pre-linguistic variables. It was the best sociodemographic variable in terms of explanatory weight. The effect of age on vocabulary growth reflects a long-established fact regarding the initial course of vocabulary learning: The transition from slow to rapid word-learning in the first half of the child’s second year [107,108]. Authors such as Bloom [107] and Nelson [108] observed a sudden increase in new word learning from the age of 17 months, which is the age at which in our data we observed an increase in the rate of vocabulary acquisition. More recently, Fenson et al. [12], using the English MacArthur–Bates CDIs, also observed a considerable gain in the 16–18 month period. Therefore, the results obtained in our study reflect the phenomenon known as the vocabulary or lexical spurt, according to which most children increase their vocabulary notably between these months, as shown by the fact that the function that best explains the rate of acquisition of vocabulary has an exponential nature. This abrupt increase in vocabulary learning can be cognitively interpreted as the acquisition of a new learning procedure. When a “critical mass” of vocabulary is reached (approximately after 50 items, independently of age), words go from being simple gestures or acoustic signals to progressively decontextualized signs. Then, new labels and later words, accepting morphological marks according to their category, are quickly incorporated [48,109,110]. Children are then said to have acquired a new learning strategy which opens the path towards full adult competence [111].

### 4.2. Sociodemographic and Pre-Linguistic Predictors of Early Vocabulary

Birth order has been associated with language development at earlier ages, with better grammatical and vocabulary skills in firstborns [13,14,30], a finding our results support. In our sample of 10–18-month-old children acquiring Catalan, firstborns show a higher vocabulary production than later-borns. The relation between vocabulary growth and birth order is probably mediated by the direct adult–child speech parents can establish, and is associated with the quantity and quality—more input received from siblings and less directly from parents—of the language received by the child [30,36,37]. However, other studies failed to find this relationship (see for example, [32]), or have found it only temporarily during development [31]. The results of Pine [31] indicated that this relationship could be stronger at the beginning of language acquisition [112] and as the child grows this relationship weakens. Previous studies have also found that second-born children show better communication skills [30] because communication depends largely on socialization, as highlighted by different authors [113]. Thus, as different authors have shown, birth order cannot be considered a risk factor of language delay [30,114]. Differences due to birth order in language development are a reflex of different language contexts where first-borns receive more direct-adult speech that improves grammar and lexical development, and later-borns receive a greater variety of conversations and communication opportunities that improve communication skills [30,114].

Birth weight was also a significant predictor of vocabulary size at 10–18 months in our study. However, its impact compared to the other variables was small, maybe because we only included healthy children with a weight over 1900 g. In fact, a low birth weight has been found to be related with medical complications, such as deafness and cerebral palsy, which could cause subsequent language and developmental difficulties [22], but these children were excluded from our study. Meanwhile, moderately low birth weight (between 1900 and 2500 g) in healthy children has shown divergent results across studies, some indicating that birth weight affects early vocabulary development and others not [24,115]. 

We failed to find a relationship between vocabulary score and the other sociodemographic factors (sex, otitis episodes, and mother’s level of education) previously related with language development in our sample of young Catalan language learners. It may be that the relationship between these sociodemographic factors and language acquisition is mediated by other variables or that they have an influence during later language development.

Sex did not predict a higher vocabulary rate in 10 to 18-month-old Catalan children when pre-linguistic variables were included in the regression model. This result is contrary to other studies of English and non-English languages [13,14,15,16]. Our data are in better agreement with a recent large-scale study that found few differences between girls and boys in language development [9]. Some researchers in language development and language disorders have stated that during the last decades, especially in clinical contexts, there has been a diagnostic bias of language difficulties regarding boys, and a misdiagnosis of girls because the latter show less evident symptoms and go unnoticed more often [34,116,117].

With regard to temporal mild hearing loss, our results are consistent with those of a meta-analysis by Roberts et al. [28], which indicated that the number of otitis episodes is not related to the variance of vocabulary production. This is a variable that was considered to be a risk factor for spoken language difficulties in clinical settings [29], but our evidence does not support this.

With respect to the mother’s education level, diverse studies have pointed out its influence in language development [33,38,43]. Nevertheless, this influence seems to be mediated by the linguistic input that the child receives [38] and the quality of parental communication (e.g., direct speech, routines…) [118]. Our data do not indicate that the mother’s education level explains any noticeable variance in early vocabulary development. Although other studies also failed to find this relation in our context [118], it is possible that this variable has a greater impact in later development.

Among prelinguistic factors, our results show that the comprehension of frequent phrases is a significant predictor of vocabulary. This fact is relevant and give rise to some reflections on how the simple fragments and phrases that conform this section (“a dormir”/“go to sleep”, “anem a banyar”/“let’s take a shower”, “fes-me un petó”/“give me a kiss”, “obre la boca”/“open your mouth”, “molt bé”/“very well”, “Què és això?”/“what is that?”, “Vols…?”/“do you want...?”, “T’has fet caca?”/“did you poop?”) are facilitators and precursors of vocabulary learning. This typical language directed at children is redundant, highly contextualized, and with overlapping clues (gestural, visual, and contextual), features that help children to analyse and recognize words and intentions. Thus, children pay attention to language not only by observing the formal composition of parental productions, but also their function, meaning, and referentiality. They profit from gathering the initial understanding of the first orders or demands, the understanding of the pragmatic intentions in the speech acts, and the exchange of questions highly contextualized about actions. In that sense, our findings are aligned with the experimental studies of Cartmill et al. [119], who showed that the quality of parental input, in particular the opportunities they offer for understanding and producing words in a contextualized and informative medium at 14 and 18 months, is a good predictor of the vocabulary level at 3 years of age. Another recent study [120] confirmed that the parent coaching in 8 and 14-month-old infants correlates with vocabulary levels at 18 months. 

Parental input is one of the best predictors of a child’s later language performance and our study has shown that the understanding of these prototypical examples of child-directed speech is related to the course of learning words. This result agrees with those that have reported a strong correlation between the comprehension of sentences in the initial years and the later language level [45,63,64]. Future studies should analyze more carefully whether the understanding of these first and repetitive adult productions, together with the acquisition of the other pre-linguistic and linguistic factors reported here, is a necessary condition for progress in vocabulary learning.

Verbal imitation was the variable with the strongest predictive power of vocabulary growth in our study. Together with Tennie et al. [121], we consider imitation central to any explanation of our complex culture. Language according to many authors is the most powerful cultural artefact transmitted from one generation to another [122]. Nevertheless, the number of studies on verbal imitation are still scarce compared to the large body of research that exists on the imitation of instrumental actions. In this study, we tried to resume the interest in the study of the role of imitation in language acquisition, specifically in the production of vocabulary during the early years of life. Our study clearly shows that, among pre-linguistic factors, verbal imitation explains the highest percentage of the variance of expressive vocabulary, as was also found in previous studies [74,75,76,80]. Children who showed more verbal imitations of their mothers’ productions at 13 months were those who at 17–21 months had more advanced vocabulary skills. 

Similar studies carried out including children with atypical development also corroborate our results. Yoder and Layton [82] found that imitation ability positively predicted the size of the initial spoken vocabulary in children with ASD. In another recent study carried out with children with ASD [83], it was observed that verbal imitation at 20 and 71 months, as evaluated by the CDI inventories, is associated with a subsequent rapid growth of expressive vocabulary. In a study conducted including children with Down syndrome, it was found that poor verbal imitation may negatively influence the extent to which words enter the child’s repertoire [81]. Therefore, imitation can be seen as a strategy that infants use for representing and encoding new verbal behaviors, and incorporating them into an existing repertoire involves reproducing and acquiring a new ’word’ in its appropriate form and function [123,124]. Imitation also can be seen as a behavior for interacting with others—it can serve to acknowledge interactions with others, maintain the topic, or take turns. Although not all children use imitation, it has the potential to advance vocabulary acquisition by facilitating the processes of mental representation, analysis, and practice of linguistic structures. As a strategy, beyond the first half of the second year it may not be as effective [108]. 

From our results it can be concluded that verbal imitation can be used to distinguish between children who are going to have good linguistic development and those who are at risk of presenting some difficulties or delay in the development of language. While our results point to a significant effect of verbal imitation in language development, in child development assessments it will be important to explore it as a warning sign to detect atypical children or those who are at-risk in their early development. Furthermore, based on the results of our study, verbal imitation can be understood as an effective pre-linguistic training strategy for professionals working with young children. Thus, in children with early language development difficulties, verbal imitation could be a strategy to favour later language development. 

In addition to imitation, our results have shown the importance of late gestures and actions as predictors for initial vocabulary. Beyond deictic gestures or emblems, it was observed that complex actions or gestures constitute the second strongest predictor of vocabulary acquisition. As described in Section 2, “later gestures” include actions of symbolic play or activities related to it. Therefore, the data obtained do not support the idea that communicative gestures or routines (“early gestures”) as a whole are adequate predictors of vocabulary acquisition in the age range we have studied. As some authors have argued [93], it is possible that they are only behavioural antecedents that prepare the emergence of expressive language. By contrast, late gestures or actions related to symbolic play are good predictors of the level of vocabulary, in line with the findings of another study [98]. Language and symbolic play reflect the development of underlying mental representative functions. Several studies concerning the relationship between early language and symbolic play have established temporal correlations in functional and structural development [99,125,126,127]. In the present study, this relationship was also found. Vocabulary acquisition is related to symbolic play, so symbolic play can predict the rate and size of the expressive vocabulary. However, more research is needed to clarify this relationship at other ages and for other language components in order to explain the causal direction of these influences.

Although the present study presents several strengths, including the joint analysis of relevant factors in language development in a large sample, it also has some limitations. One of the most important limitation is the use of a cross-sectional analysis instead of a longitudinal analysis. Future studies would benefit from following children through their development to show how these variables influence the full developmental language process. Another aspect that must be further studied is the time that parents stay at home with their children, whether they work, and whether the child attends preschool centers. These data would allow us to gain a broader overview of the factors related to learning vocabulary. 

Our results have several practical implications. First, they highlight that although sociodemographic variables play a role in identifying early language difficulties, they cannot be used alone to detect children at risk and must be combined with other prelinguistic risk factors. Second, our results provide further evidence that the presence of verbal imitations and symbolic play in the first two years of life are indicators of a positive prognostic of language development. In this sense, a greater number of risk factors seem to increase the probability of language delay [128,129]. We recommend that the progress of those children who show low levels of imitation and restricted symbolic play during their first and second years should be followed closely, especially if they were born with a low birth weight and are not the firstborn. It would also be recommended to promote imitation and symbolic play through language interventions delivered in naturalistic contexts (home or kindergarten preschool teachers or parents). In this sense, language interventions based on imitation and symbolic play have shown effective results in children with language difficulties [130,131,132]. Nevertheless, it is important to note that associated risk factors may differ depending on the age of the child and may change as children develop [128]. 

## 5. Conclusions

In this study we have shown that the relationship between pre-linguistic abilities and vocabulary competence is strong and that the former can predict either normal or delayed progress. Although the age, birth order and birth weight of the child are related to vocabulary size, these sociodemographic (non-linguistic) factors have low explanatory power and cannot be used in isolation as an early warning sign for vocabulary delays. The imitation of words or statements, participation in symbolic play activities, and the understanding of highly contextualized phrases are powerful predictors that identify the linguistic functions, the meanings and use of the first words of children and, thus, aid their learning. These results are highly relevant and helpful in early child communication and language development, either for the prevention of difficulties and, if necessary, for early interventions.

## Figures and Tables

**Table 1:** Main descriptive data of the participants.

Personal and Sociodemographic Characteristics	*N* = 504
Age in months, *M* (SD)	14.23 (2.5)
Sex as % female	51.4
Birth weight in kg, *M* (SD)	3.26 (0.48)
Number of ear infections per year, *M* (SD)	0.52 (1.29)
Birth order in % of children	
First	56.8
Second	36.8
Third	5.4
Fourth, onwards	1
Mother’s educational level, %	
No studies	0.2
Primary	4.4
Secondary	29
University	66.4
Father’s educational level, %	
No studies	0.2
Primary	12.3
Secondary	40.9
University	46.6

Note: *M* = mean; SD = standard deviation.

**Table 2:** Descriptive data on language precursors.

Language Precursors	*N* = 504
First signs of understanding, %	
0 or 1 behaviour	0.8
2 behaviours	10.6
3 behaviours	88.6
Imitation, %	
Not yet	40.9
Sometimes	41.9
Often	17.2
Phrases, *M* (SD)	17.93 (7.09) ^a^
First gestures, *M* (SD)	14.73 (4.12) ^b^
Late gestures, *M* (SD)	17.02 (9.52) ^c^

^a^ Maximum score: 27, ^b^ Maximum score: 25, ^c^ Maximum score: 44.

**Table 3:** Multivariate regression analysis of expressive vocabulary with respect to potential pre-linguistic and sociodemographic predictors.

Predictors	Coefficient (95% CI)	*β*	*p*
Age	0.1 (0.05 to 0.15)	0.167	<0.001
Birth weight	0.19 (0.02 to 0.36)	0.063	0.031
Birth order	−0.15 (−0.28 to −0.02)	−0.081	0.021
Imitation	0.85 (0.73 to 0.98)	0.445	<0.001
Late gestures and actions	0.04 (0.03 to 0.05)	0.245	<0.001
Phrases	0.03 (0.01 to 0.04)	0.140	0.002

CI indicates confidence interval; *R*^2^ = 0.656; Adj. *R*^2^ = 0.651.

**Table 4:** Multivariate logistic regression analysis of large vocabulary.

Predictors	OR (95% CI)	*p*
First model		
Imitation	6.889 (4.455 to 10.651)	<0.001
Second model		
Imitation	5.348 (3.400 to 8.411)	<0.001
Late gestures and actions	1.067 (1.036 to 1.099)	<0.001

CI, confidence interval; *N* = 428 (76 missing values); *R*^2^ Nagelkerke: 0.374; OR = Odds ratio.

**Table 5:** Specificity and sensitivity of the model.

	Observed Percentile		
Predicted percentile	<25	>25	
<25	69	50	
>25	59	250	
	Sensitivity	Specificity	Accuracy
	53.9%	83.3%	74.5%

## Appendix A

**Table A1:** Statistics from the stepwise regression model.

Model	Predictors	Coefficient (95% CI)	*β*	*p*	AdjR^2^
1	Imitation	1.31 (1.18 to 1.45)	0.677	<0.001	0.456
2	Imitation	0.94 (0.82 to 1.07)	0.486	<0.001	0.622
	Late gestures and actions	0.07 (0.06 to 0.08)	0.450	<0.001	
3	Imitation	0.90 (0.78 to 1.02)	0.464	<0.001	0.637
	Late gestures and actions	0.05 (0.04 to 0.06)	0.327	<0.001	
	Age	0.11 (0.06 to 0.16)	0.183	<0.001	
4	Imitation	0.87 (0.74 to 0.99)	0.477	<0.001	0.645
	Late gestures and actions	0.04 (0.03 to 0,05)	0.266	<0.001	
	Age	0.08 (0.03 to 0.13)	0.142	0.001	
	Phrases	0.03 (0.01 to 0.05)	0.139	0.001	
5	Imitation	0.86 (0.74 to 0.99)	0.445	<0.001	0.648
	Late gestures and actions	0.04 (0.03 to 0.05)	0.270	<0.001	
	Age	0.09 (0.04 to 0.14)	0.154	0.001	
	Phrases	0.03 (0.01 to 0.04)	0.129	0.003	
	Birth order	−0.14 (−0.27 to −0.01)	−0.063	0.032	
6	Imitation	0.85 (0.73 to 0.98)	0.445	<0.001	0.651
	Late gestures and actions	0.04 (0.03 to 0.05)	0.245	<0.001	
	Age	0.1 (0.05 to 0.15)	0.167	<0.001	
	Phrases	0.03 (0.01 to 0.04)	0.140	0.002	
	Birth order	−0.15 (−0.28 to −0.02)	−0.081	0.021	
	Birth weight	0.19 (0.02 to 0.36)	0.063	0.031	
